# A Rare Presentation of Patella Button Aseptic Loosening After a Total Knee Replacement Without Evidence of Radiographic Loosening

**DOI:** 10.7759/cureus.34665

**Published:** 2023-02-06

**Authors:** Hak Lian Teh, Ahmad Fauzey Kassim, Suresh Chopra, Veenesh Selvaratnam

**Affiliations:** 1 Exeter Hip Unit and Exeter Knee Reconstruction Unit, Princess Elizabeth Orthopaedic Centre, Royal Devon and Exeter National Health Service Foundation Trust, Exeter, GBR; 2 Arthroplasty Unit, Sultanah Bahiyah Hospital, Alor Setar, MYS; 3 National Orthopaedic Centre of Excellence in Research and Learning, Department of Orthopaedic Surgery, Faculty of Medicine, Universiti Malaya, Kuala Lumpur, MYS

**Keywords:** three-peg patella button, anterior knee pain, total knee replacement, patella button loosening, patella resurfacing

## Abstract

Patella resurfacing in total knee replacement (TKR) has been shown to reduce the rate of anterior knee pain, but there are complications from patella resurfacing. A 54-year-old male underwent a left primary TKR with patella resurfacing 15 years ago. He developed spontaneous progressive anterior knee pain for six months. At revision surgery, his patella button was found to be loose. Loosening of a three-peg patella button is rare. A high index of suspicion of patella button loosening should be suspected in patients who present with anterior knee pain after patella resurfacing.

## Introduction

Total knee replacement (TKR) remains a cost-effective procedure in moderate-to-severe knee osteoarthritis. It is also an excellent surgery to relieve pain secondary to knee osteoarthritis. The need for patella resurfacing in conjunction with a TKR remains debatable [1,2]. The Australian and the United Kingdom joint registries have shown an increased risk of revision if the patella was not resurfaced, especially with a posterior stabilized (PS) implant, while other joint registries such as the Norwegian and Swedish registries showed no difference [3,4].

Patella resurfacing is proven to reduce the prevalence of anterior knee pain following TKR; however, complications such as patella subluxation, maltracking, fracture, extensor mechanism failure, and implant loosening do arise secondary to resurfacing. However, loosening followed by implant dissociation and extra-articular migration is extremely rare [5,6].

We report a case of atraumatic patella button loosening after 15 years of initial uncomplicated TKR, which is exceedingly rare. There was no evidence of osteolysis from the preoperative imaging to suggest a loose patella button.

## Case presentation

We report a 56-year-old male who had an uneventful primary left TKR (Scorpio PS NRG, Stryker, Kalamazoo, MI, USA) performed 15 years ago at a different center. He presented to our center with left anterior knee pain for the past six months. He was dependent on a walking stick due to his pain. The patient has a past medical history of ischemic stroke five years ago with no residual weakness. 

His examination demonstrated a well-healed midline scar with minimal effusion. He had a range of motion of 5°-120° with severe tenderness, especially on the lateral side of his patella facet. He had a positive Clarke’s test. His preoperative Oxford Knee Score (OKS) was 22.

**Figure 1:**
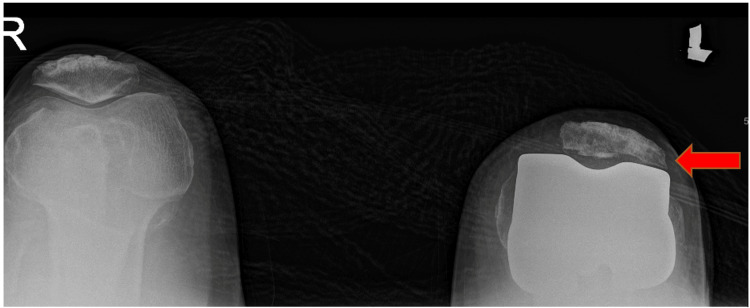
Skyline view of the patella with impingement of the left lateral patella facet (arrow) with no obvious osteolysis or loosening of the patella component

**Figure 2:**
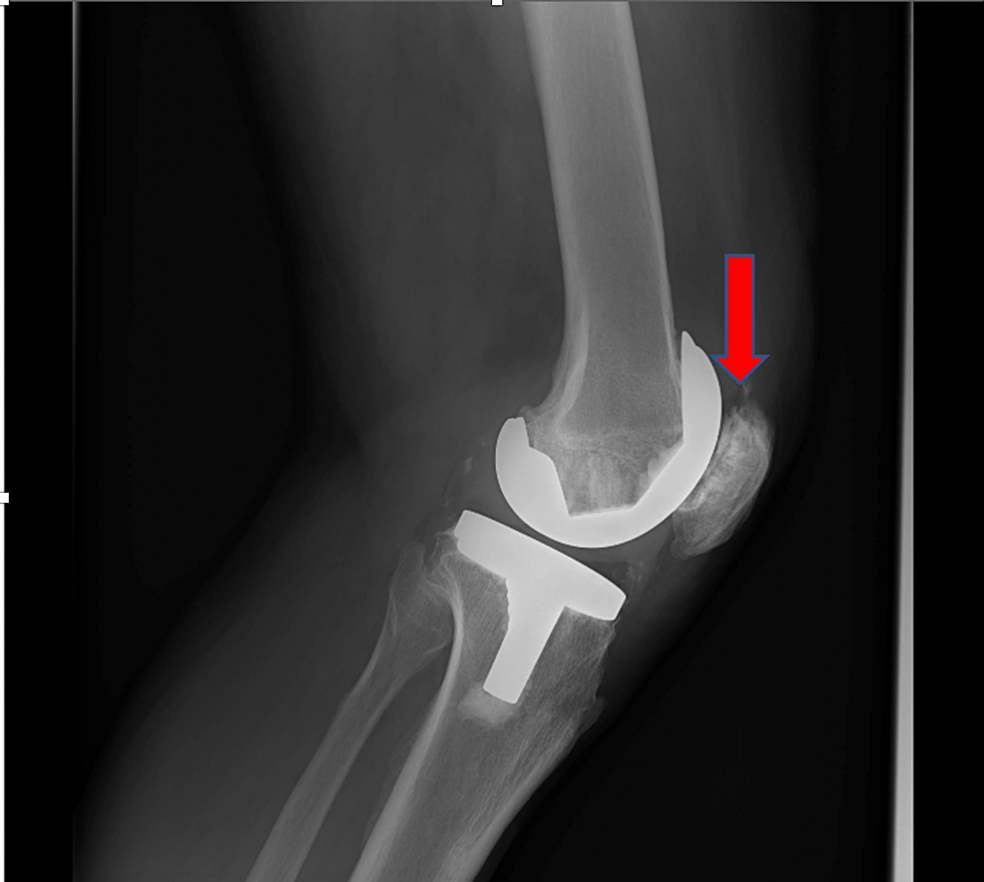
A well-fixed left TKR with no evidence of patella button loosening (arrow) TKR: total knee replacement

Preoperatively, infection markers were negative, and an X-ray showed possible impingement of the lateral patella facet on the femur implant in the skyline view (Figure 1). There was no evidence of patella button loosening, and all components looked well fixed (Figure 2).

**Figure 3:**
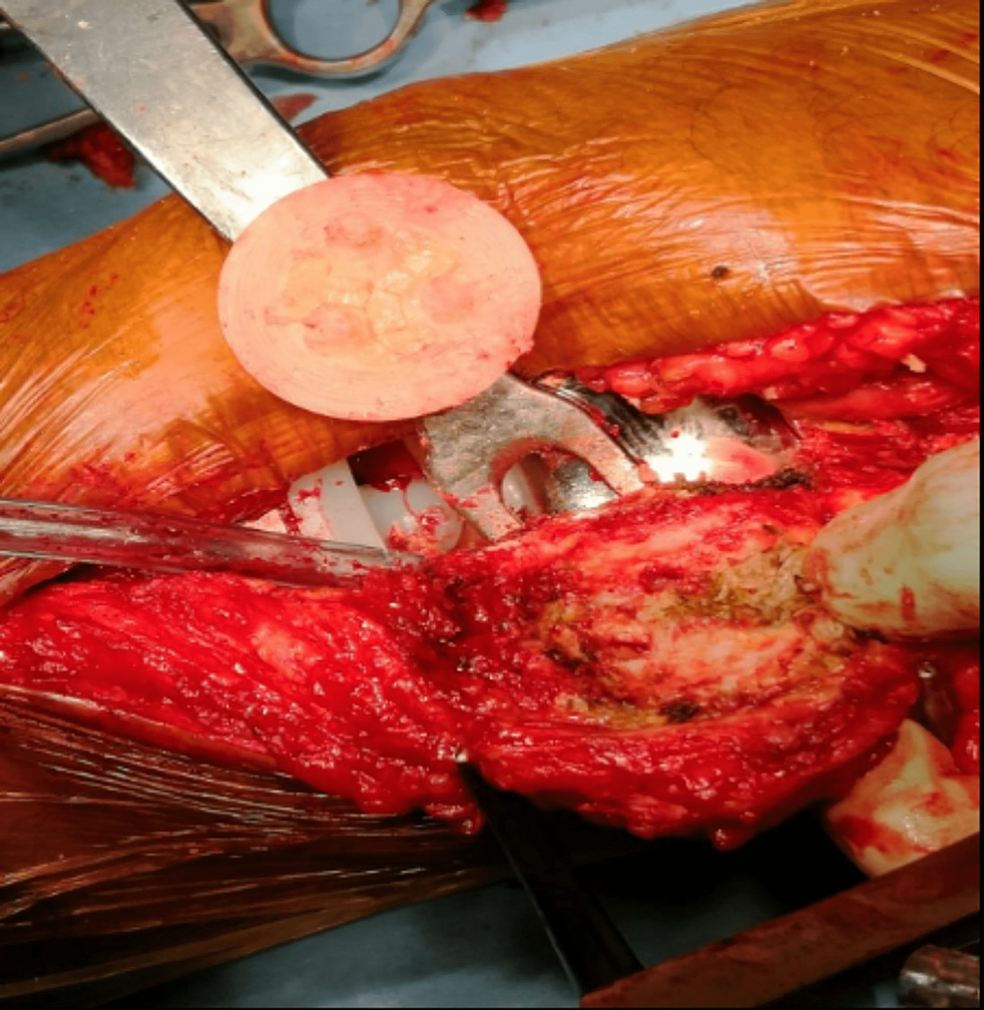
A loose patella button was noticed after everting the patella intraoperatively; the femoral and tibial components were well fixed

**Figure 4:**
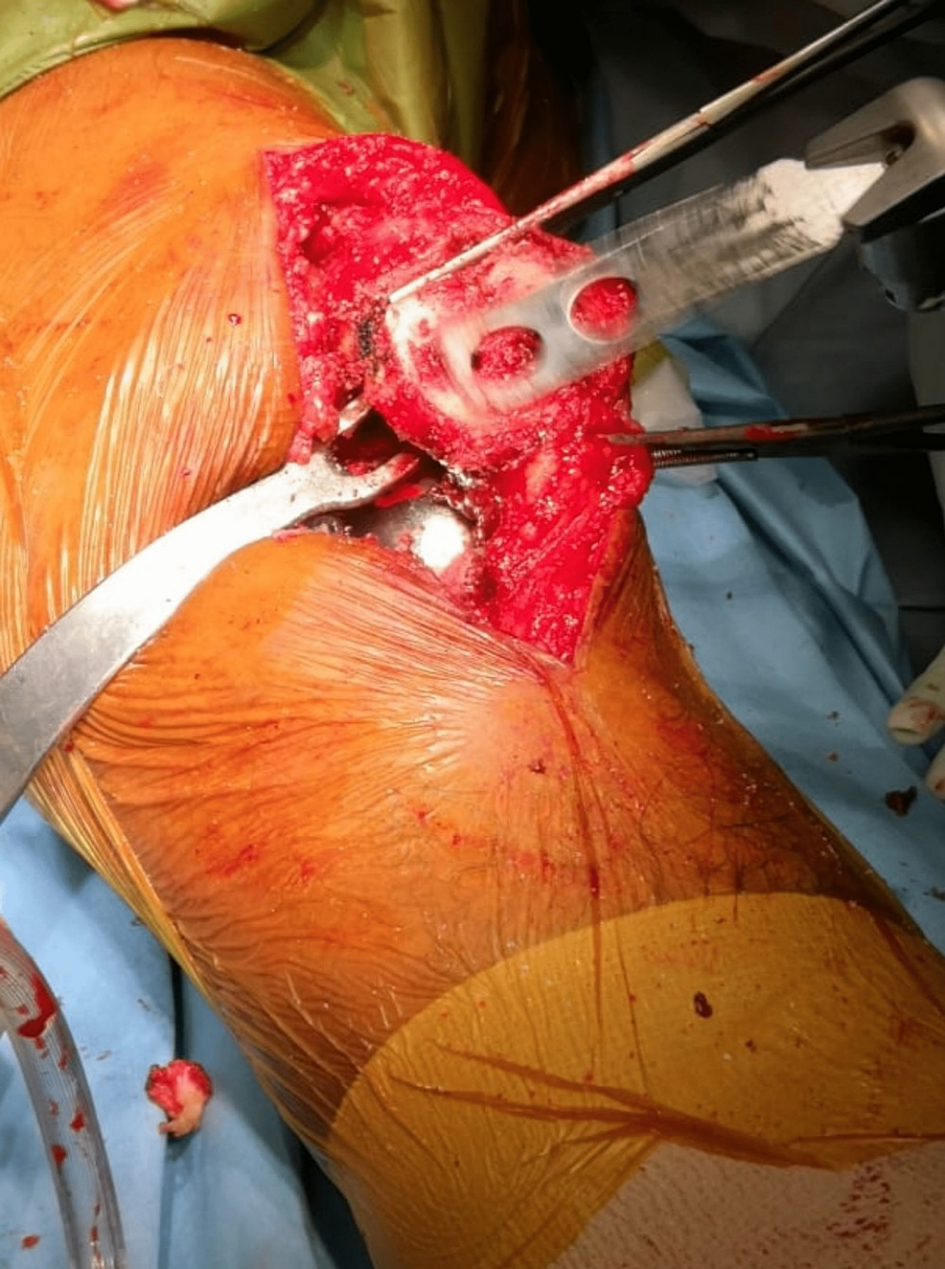
Lateral patella facet decompression and freshening patella cut was performed prior to patella re-resurfacing

The preoperative plan was to perform a decompression of the lateral patella facet, which was most probably the cause of his anterior knee pain. Intraoperatively, we found that the three-peg hole patella button was loose and not attached to the inner surface of the patella (Figure 3). Therefore, in addition to lateral patella facet decompression (Figure 4), re-resurfacing of the patella was carried out. The patella bed was cleared of excess membrane and cement with a freshening saw cut. The remaining patella thickness was 12 mm with a good bleeding bone bed. A patella re-resurfacing using a cemented symmetrical 31 mm by 9 mm three-peg hole patella onlay button was performed.

**Figure 5:**
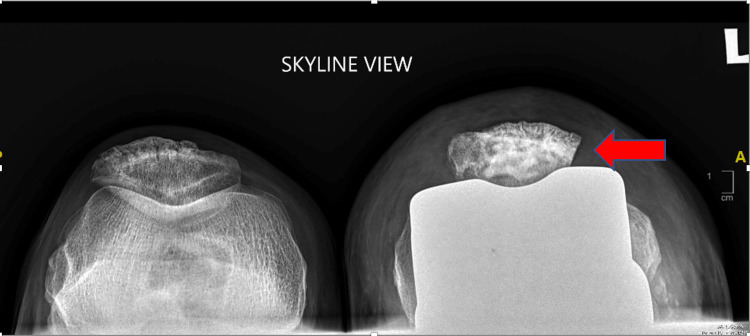
Postoperative skyline view demonstrating the left knee lateral patella facetectomy (arrow) and a well-seated patella re-resurfacing

**Figure 6:**
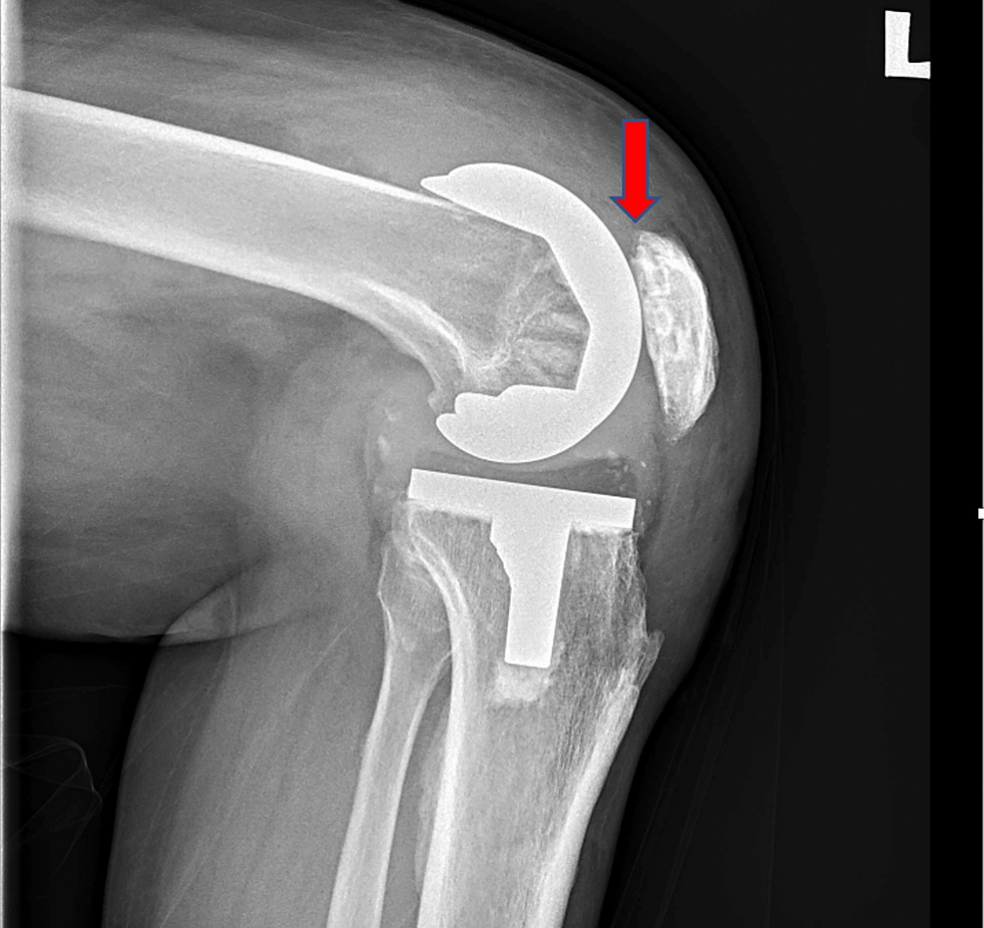
Lateral radiograph of the knee showing well-seated implants and patella re-resurfacing (arrow)

The patient was discharged the next day postoperatively after satisfactory knee radiographs (Figure 5 and Figure 6) and three doses of IV antibiotics. His pain improved significantly when he was seen in subsequent clinic follow-ups. He is currently independently mobile, and his OKS at one-year post-re-resurfacing of the patella was 46.

## Discussion

Daily range of movement of the knee results in shearing coronal forces as well as compressive sagittal forces across the patellofemoral joint (PFJ). The magnitude of the joint reaction force (JRF) increases as the knee flexes to maintain equilibrium against rising quadriceps force and patella tension [7,8]. The JRF during flexion increases fourfold compared to during extension [9]. There is a lateral shear force acting on the patella button between 0° to 50°; this shear force changes its direction to the medial side as the knee flexes from 55° to 90° [10]. These changes in the direction of the articular shear force result in a cyclic rocking effect on the cement peg interface in a medial-lateral fashion [8]. These forces and the changes that occur during extension and flexion are transmitted to the cement implant interface both in the coronal and sagittal planes, causing patella component loosening [8]. The design of the patella is crucial to reduce and accommodate these stresses. The features in the patella button, such as peg indentations and cement recess, as well as good cementation and pressurization technique, may improve the cement fixation of the patella button.

Anterior knee pain post-TKR is multifactorial. It can be caused by component malrotation [11] and other various patellofemoral complications, such as implant maltracking, patellar fracture, aseptic loosening, and polyethylene wear [12,13]. The decision on whether to resurface the patella remains controversial. Parvizi et al. [14] found that patients who did not have their patella resurfaced during TKR had significantly greater anterior knee pain. Appropriately 8.7% had to eventually need further unplanned surgery for secondary resurfacing. The patellar component is frequently positioned parallel to the anterior cortex of the patella and slightly medialized to reduce the quadriceps angle (Q angle) and help with patella tracking. This will inadvertently leave the lateral patella facet exposed. This exposed lateral facet can be removed by making a perpendicular saw cut to the surface of the patella or using a nibble to nibble the exposed lateral patella facet. This will decompress the patella and help with patella tracking.

Yuenyongviwat et al. [15] in their cadaveric study showed a significant decrease in patellofemoral contact pressure after lateral patella facetectomy. Similarly, Zhang et al. [16] stated that lateral facetectomy in non-patella resurfaced TKR decreased the mismatch of the femoral component and lateral facet of the patella. It also reduces the tension at the lateral retinaculum. This results in a patella that is more congruent with the femoral component.

Our patient had an elongated lateral patella facet that was impinging on the lateral femoral component, which is the root cause of severe lateral facet pain. We initially thought that this could be managed by lateral patella facetectomy alone, after excluding other causes of anterior knee pain. Unfortunately, his patella button was found to be loose and totally disengaged from the patella surface intra-operatively. Therefore, patella re-resurfacing was performed in addition to lateral patella facetectomy. Patella button dissociation is an uncommon complication. Failure of the patella component in total knee replacement, especially aseptic loosening of the patella button, tends to occur earlier [17,18].

Standard radiographs of the knee such as anterior-posterior, lateral, and skyline views are routinely obtained as initial radiographs to investigate problematic TKRs. The skyline and lateral views are often performed with the knee flexed. This compressive force results in the patella button being compressed between the trochlear component and the cement mantle. Therefore, the signs of loosening on plain radiographs may be masked, even though there is loosening in the cement implant interface. This was demonstrated in our case. Our patient’s inflammatory markers and synovial fluid analysis were negative preoperatively. We could have performed a preoperative arthroscopic assessment of the knee, but we have a high threshold of performing this due to the risk of infection.

## Conclusions

In conclusion, lateral patella facetectomy should be performed, if possible, to decompress the anterior-lateral compartment and reduce the risk of impingement. Aseptic loosening of the patella button tends to occur earlier as reported by a few authors, but surgeons should also have a high index of suspicion in cases of late presentation.

## References

[1] Murray DW, MacLennan GS, Breeman S (2014). A randomised controlled trial of the clinical effectiveness and cost-effectiveness of different knee prostheses: the Knee Arthroplasty Trial (KAT). Health Technol Assess.

[2] Evans JT, Walker RW, Evans JP, Blom AW, Sayers A, Whitehouse MR (2019). How long does a knee replacement last? A systematic review and meta-analysis of case series and national registry reports with more than 15 years of follow-up. Lancet.

[3] Ben-Shlomo Y, Blom A, Boulton C (2022). The National Joint Registry 19th Annual Report 2022 [Internet]. National Joint Registry 19th Annual Report.

[4] Australian Orthopaedic Association National Joint Replacement Registry (AOANJRR) (2021). Hip, knee & shoulder arthroplasty: 2021 annual report. Hip, Knee & Shoulder Arthroplasty Annual Report.

[5] Meding JB, Fish MD, Berend ME, Ritter MA, Keating EM (2008). Predicting patellar failure after total knee arthroplasty. Clin Orthop Relat Res.

[6] Jacobs E, Feczko P, Emans P (2013). Recurrent patella loosening and extra-articular migration after TKA. J Knee Surg.

[7] Amis AA, Senavongse W, Darcy P (2005). Biomechanics of patellofemoral joint prostheses. Clin Orthop Relat Res.

[8] Rath NK, Dudhniwala AG, White SP, Forster MC (2012). Aseptic loosening of the patellar component at the cement-implant interface. Knee.

[9] Huberti HH, Hayes WC (1984). Patellofemoral contact pressures. The influence of q-angle and tendofemoral contact. J Bone Joint Surg Am.

[10] Browne C, Hermida JC, Bergula A, Colwell CW Jr, D'Lima DD (2005). Patellofemoral forces after total knee arthroplasty: effect of extensor moment arm. Knee.

[11] Berger RA, Crossett LS, Jacobs JJ, Rubash HE (1998). Malrotation causing patellofemoral complications after total knee arthroplasty. Clin Orthop Relat Res.

[12] Petersen W, Rembitzki IV, Brüggemann GP, Ellermann A, Best R, Koppenburg AG, Liebau C (2014). Anterior knee pain after total knee arthroplasty: a narrative review. Int Orthop.

[13] Johnson DP, Eastwood DM (1992). Patellar complications after knee arthroplasty. A prospective study of 56 cases using the Kinematic prosthesis. Acta Orthop Scand.

[14] Parvizi J, Rapuri VR, Saleh KJ, Kuskowski MA, Sharkey PF, Mont MA (2005). Failure to resurface the patella during total knee arthroplasty may result in more knee pain and secondary surgery. Clin Orthop Relat Res.

[15] Yuenyongviwat V, Iamthanaporn K, Hongnaparak T (2017). Lateral facetectomy decreased patellofemoral contact pressure in total knee replacement: a cadaveric study. J Clin Orthop Trauma.

[16] Zhang LZ, Zhang XL, Jiang Y, Wang Q, Chen YS, Shen H (2012). Lateral patellar facetectomy had improved clinical results in patients with patellar-retaining total knee arthroplasty. J Arthroplasty.

[17] Berend ME, Ritter MA, Keating EM, Faris PM, Crites BM (2001). The failure of all-polyethylene patellar components in total knee replacement. Clin Orthop Relat Res.

[18] Huang CH, Lee YM, Lai JH, Liau JJ, Cheng CK (1999). Failure of the all-polyethylene patellar component after total knee arthroplasty. J Arthroplasty.

